# 14-Day Repeated Intraperitoneal Toxicity Test of Ivermectin Microemulsion Injection in Wistar Rats

**DOI:** 10.3389/fvets.2020.598313

**Published:** 2020-12-16

**Authors:** Zhen Dong, Shou-ye Xing, Ji-yu Zhang, Xu-zheng Zhou

**Affiliations:** ^1^Lanzhou Institute of Husbandry and Pharmaceutical Sciences, Chinese Academy of Agricultural Sciences (CAAS), Lanzhou, China; ^2^Key Laboratory of Veterinary Pharmaceutical Development, Ministry of Agriculture, Lanzhou, China; ^3^Key Laboratory of New Animal Drug Project of Gansu Province, Lanzhou, China; ^4^China Agricultural Vet. Bio. Biomedical Co., Ltd, Tianjin, China

**Keywords:** ivermectin, microemulsion, rats, toxicity, 14-day, injection

## Abstract

To evaluate the safety of ivermectin microemulsion injection, 100 Wistar rats were injected intraperitoneally at 0.38 g/kg, 0.19 g/kg, and 0.1 g/kg for 14 days. The 14-day repeated toxicity test of ivermectin microemulsion injection was systematically evaluated by clinical observation, organ coefficient, hematological examination, clinical chemistry examination, and histopathological examination. The results showed that no rats died during the test. At the initial stage of treatment, the rats in the high dose group had mild clinical reaction, which disappeared after 4 days. Clinical chemistry showed that the high dose of ivermectin microemulsion could cause significant changes in ALT and LDH parameters in male rats; high and medium doses could increase the liver coefficients of male and female rats. The toxic target organ may be the liver as indicated by histopathological findings. No significant toxic injury was found in the heart, liver, spleen, lung, kidney, brain, ovary, and testes of all groups of rats. No drug-related toxic effects were found at low doses, and thus the NOVEL of ivermectin microemulsion injection was 0.19 g/kg.

## Introduction

Ivermectin (IVM) is a new macrolide antibiotic insect repellent, which has the advantages of high efficiency, broad spectrum and low toxicity. It is the first Avermectin (AVM) drug derivative treated by Merck Company of the United States. Ivermectin can be used to kill nematodes and other endoparasites with an effect of 94–100% (1, 2). It also has a good killing effect on the larvae of *Gastrophilus intestinalis, Oestrus ovis*, and the other ectoparasites, such as *Psoroptidae, Linognathidae Enderlein, Sarcoptes scabiei, Haematopinus suis* in bovine, ovis and swine (3–5). It is a safe and ideal drug for repelling endoparasites and ectoparasites, which is the most widely used in animal production. The mechanism of its action is that IVM blocked the transmission between intermediate neurons and excitatory motor neurons in the ventral nerve cord of parasites. It also inhibits protein transmission between inhibitory motor neurons and muscles, but has little effect on excitatory neuromuscular transmission (6).

A variety of dosage forms have been developed for clinical use by veterinarians, such as tablets, injections and so on (7, 8). However, the IVM preparation used in veterinary clinic has some disadvantages, such as short maintenance time of each administration, repeated administration and so on, and the blood concentration of common preparation fluctuates greatly. The phenomenon of “peak and valley” often occurs, which leads to its low safety and effectiveness. IVM is a fat-soluble substance, almost insoluble in water and soluble in many organic solvents, such as chloroform, methanol, ethanol, dichloromethane, and so on (9). In order to solve the problem of solubility, previous studies have successfully developed a new preparation of ivermectin microemulsion to achieve the solubilization of ivermectin with oil-in-water (O/W) (10). Compared with conventional injection, the fluidity of the microemulsion is better, the clinical use is more convenient, especially in the pastoral area with high altitude, and the stimulation of the formulation is less (11, 12). Intramuscular injection of 0.2 mg/kg of ivermectin microemulsion resulted in C_max_ of 70.34 ± 1.89 ng/mL, T_max_ of 3.99 ± 0.20 h, and absolute bioavailability of 91.33% in sheep; In cattle, C_max_ was 121.96 ± 4.12 ng/mL, T_max_ was 5.52 ± 0.20 h, and absolute bioavailability was 75.42% (unpublished).

However, information about the toxicity of IVM microemulsions is very limited. The purpose of this study is to evaluate and obtain the toxicological characteristics of IVM microemulsion injection, so as to provide safety guarantee for guiding clinical use.

## Materials and Methods

### Test Drug

Ivermectin microemulsion injection (1 g/100 mL) developed independently by Lanzhou institute of husbandry and pharmaceutical sciences, Chinese Academy of Agricultural Sciences. Test drug was sterilized by circulating steam and diluted with sterilized normal saline before use. Ivermectin microemulsion is a O/W form composed of ivermectin, tween 80, cremophor RH-40, 1,2-Propanediol, PEG 400, ethyl oleate (cis-9-Octadecenoic acid, ethyl ester) and water.

Physicochemical properties of ivermectin microemulsion: viscosity is 7.13 ± 0.06 mm^2^·s^−1^, conductivity is 144.00 ± 2.31/ms^−1^, refractive index is 1.37 ± 0.01, pH is 6.98~7.13, and the average particle size is 70.0 ± 2.3/nm. The microemulsion stored for a long time can remain transparent, no delamination, no drug precipitation, and high temperature, low temperature and light can remain stable.

### Animals

100 SPF Wistar rats, weighing 180–220 g, in which 50% of the sample size are either males or females, were purchased from the Experimental Animal Centre of Lanzhou General Hospital of Lanzhou military region (license No.: SCXK (Army) 2012-0020. Feeding environment: temperature is 20–26°C, relative humidity is 40–70% and animals were under a 12 h light-dark circle. During feeding, all animals have unrestricted access to food and water. The clinical health was observed for 7 days before the trial. This study has been approved by the Animal Ethics Committee of Lanzhou institute of husbandry and pharmaceutical science before starting.

### 14-Day Repeated Intraperitoneal Toxicity Test

100 Wistar rats were randomly divided into 5 groups [high dose group, medium dose group, low dose group, solvent group (ivermectin-free microemulsion) and normal saline group] with 20 rats in each group. There are 10 female and 10 male rats in each group. The results of previous studies have shown that the LD_50_ of intraperitoneal injection of IVM is 3.8256 g/kg (12). The doses were 0.38 g/kg·BW (1/10 LD_50_), 0.19 g/kg·BW (1/20 LD_50_) and 0.1 g/kg·BW [500× clinical dose (0.0002 g/kg·BW)], respectively. The preparations of each dose group and control group were mixed with appropriate concentration before each experiment. The corresponding solution 0.5 ml per rat was injected intraperitoneally every day. During the experiment, the toxicity reactions of each animal in each dose group were observed before and after daily administration, including feeding, drinking, respiration, feces, spirit, and death, the poisoning symptoms were recorded, the autopsy of the dead animals was carried out in time, and the pathological changes were recorded. Weight changes were measured and recorded every week.

### Hematology and Clinical Chemistry Examination

At the end of the experiment, all rats were fasted overnight for more than 12 h, injected with pentobarbital sodium solution, and euthanized by taking blood from the heart. The blood samples collected in the EDTAK_2_-coated tubes were analyzed using Mindray BC-2800 Vet Automatic blood analyser (MINDRAY Medical International Co., Ltd). The parameters of leukon [white blood cell count (WBC)], erythron [red blood cell count (RBC), Hemoglobin (HGB), red blood cell specific volume (HCT), mean red blood cell volume (MCV), mean corpuscular hemoglobin (MCH), and mean red blood cell hemoglobin concentration (MCHC)] and coagulation system [platelet (PLT)] were analyzed and recorded. Indicators of liver and kidney function, such as aspartate aminotransferase (AST), alanine aminotransferase (ALT), alkaline phosphatase (ALP), total bilirubin (TBIL), direct bilirubin (DBIL), total protein (TP), albumin (ALB), lactate dehydrogenase (LDH), creatine kinase (CK), creatinine (CREA), uric acid (UA), creatinine (GLU), triglyceride (TG), how-density lipoprotein cholesterol HDLC, low-density lipoprotein cholesterol (LDLC), total cholesterol (TCH), α-hydroxybutyrate dehydrogenase (HBDH), and urea nitrogen (BUN). Erba XL-640 Automatic biochemical analyser (Erba Mannheim Co., Ltd.) was used for clinical chemistry examination.

### Organ Coefficient and Histopathological Examination

After the end of the test, the rats in each dose group were killed, and the main organs were examined immediately to observe whether the main organs and glands were visible to the naked eye, such as swelling, shrinkage, color, hardness, and so on. The heart, liver, spleen, lung, kidney, ovary, testis, and brain were taken out and weighed, respectively, and the organ coefficients of each organ were calculated. The calculation formula is as follows:

$$\begin{array}{l} \text{Organ coefficient }=\frac{\text{organ weight}}{\text{body weight}} \end{array}$$

After that, heart, liver, spleen, lung, kidney, ovary, testis, brain, and other organs that have been weighted were fixed with 10% neutral formalin solution, routine paraffin-embedded sections, HE staining, microscopic examination, and microphotography.

### Statistical Analysis

The data were expressed as mean ± standard deviation, and the significance of the data between groups was tested by SPSS 26.0 software (IBM, Chicago, USA). The data involved in the experiment were tested for unevenness and variance, and if the hypothesis was accepted, one-way ANOVA test was used to compare the significant differences in average weight gain, hematology and clinical chemistry parameters between the treatment group and the control group. Conversely, the data will be analyzed using the non-parametric test. The significance was expressed by *P* < 0.05.

## Results

### General Observation and Weight Gain

**Figure 1:**
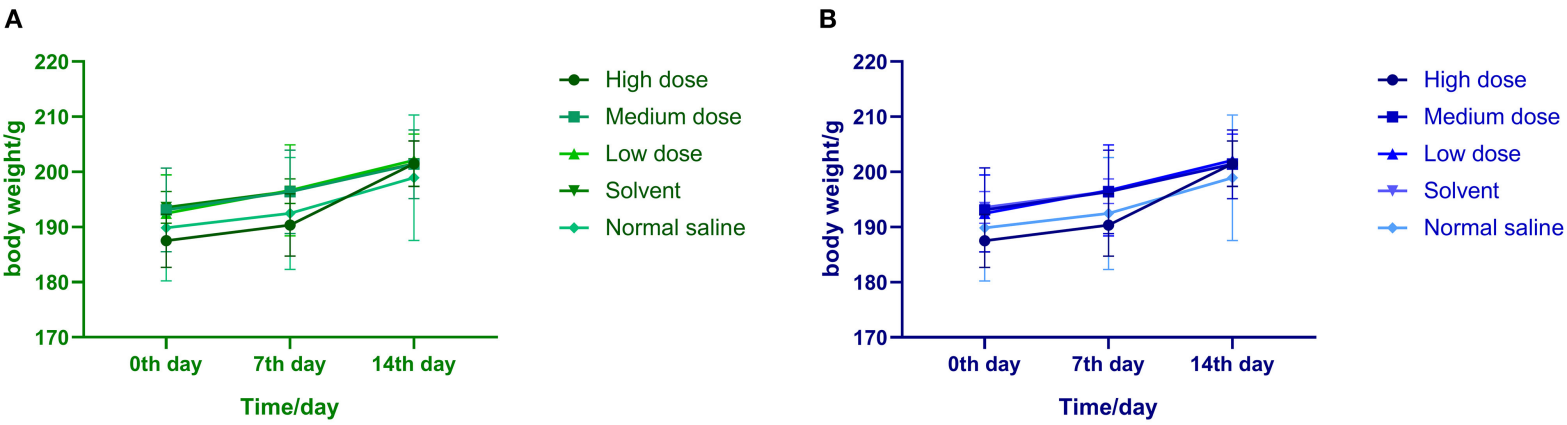
**(A)** Effects of ivermectin microemulsion injection on the body weight of male rats in each group. **(B)** Effects of ivermectin microemulsion injection on the body weight of female rats in each group.

After intraperitoneal injection of ivermectin microemulsion, slight clinical reactions such as sluggish, curled up, slow movement and disheveled coat were observed in the high dose group at the beginning, but the symptoms disappeared 4 days later. The rats in each group ate and drank normally, their mental state was good, and there was no death. There was no significant difference in weight gain among the five groups at each time point, as shown in Figure 1. There is no significant difference between the two sexes.

### Hematology and Clinical Chemistry

#### Hematology

WBC of male rats in the high dose group was significantly lower than that in the normal saline group and the solvent group (*P* < 0.05). No significant differences in other indicators. The effects of high doses of ivermectin microemulsion injection were more pronounced in female rats. Significant reductions of WBC, RBC, HGB, and HCT were seen in female rats in the high dose group compared to the normal saline group. The results of effects of ivermectin microemulsion on hematology in rats were shown in Tables 1 and 2.

**Table 1:** Effects of ivermectin microemulsion on hematology in male rats.

**Parameters**	**High dose**	**Medium dose**	**Low dose**	**Solvent**	**Normal saline**
WBC (× 10^9^·L^−1^)	8.73 ± 2.09^#,*^	13.53 ± 1.60	15.33 ± 1.70^#^	12.85 ± 2.57	13.42 ± 1.01
RBC (× 10^12^·L^−1^)	9.31 ± 0.41	9.22 ± 0.40	11.01 ± 1.11	9.75 ± 2.09	9.43 ± 0.15
HGB (g·L^−1^)	180.50 ± 8.04	174.67 ± 9.50	212.00 ± 20.50	186.00 ± 40.34	181.17 ± 2.40
HCT	0.57 ± 0.02	0.55 ± 0.03	0.66 ± 0.07	0.58 ± 0.12	0.57 ± 0.01
MCV (f L)	61.35 ± 1.38	59.85 ± 0.96	60.10 ± 0.80	59.98 ± 1.70	60.45 ± 2.05
MCH (p g)	19.43 ± 0.26	18.93 ± 0.26	19.27 ± 0.35	19.08 ± 0.28	19.22 ± 0.28
MCHC (g·L^−1^)	317.17 ± 1.60	316.50 ± 5.28	320.50 ± 4.04	317.67 ± 4.59	317.50 ± 6.38
PLT (× 10^9^·L^−1^)	1252.25 ± 89.21	1266.67 ± 53.94	1143.60 ± 31.38	1235.00 ± 53.20	1297.25 ± 42.80

* VS normal saline (P < 0.05), and

# *VS solvent (P < 0.05)*.

**Table 2:** Effects of ivermectin microemulsion on hematology in female rats.

**Parameters**	**High dose**	**Medium dose**	**Low dose**	**Solvent**	**Normal saline**
WBC (× 10^9^·L^−1^)	7.86 ± 1.45^#,*^	9.03 ± 1.29	9.47 ± 0.94	11.23 ± 2.67	10.05 ± 0.89
RBC (× 10^12^·L^−1^)	8.30 ± 0.39^#^	8.82 ± 0.44	8.53 ± 0.43	8.76 ± 0.27	8.63 ± 0.24
HGB (g·L^−1^)	161.00 ± 7.95^#^	173.50 ± 8.60	164.50 ± 6.53	170.67 ± 4.80	168.33 ± 4.84
HCT	0.51 ± 0.02^#^	0.54 ± 0.03	0.52 ± 0.02	0.54 ± 0.02	0.53 ± 0.02
MCV (f L)	61.18 ± 0.58	61.32 ± 0.35	61.25 ± 0.92	61.38 ± 1.16	60.67 ± 0.30
MCH (p g)	19.43 ± 0.26	19.68 ± 0.17	19.30 ± 0.42	19.50 ± 0.42	19.52 ± 0.26
MCHC (g·L^−1^)	317.17 ± 1.60^*^	320.83 ± 2.64	315.17 ± 3.92^#^	317.33 ± 4.13^#^	321.83 ± 3.49
PLT (× 10^9^·L^−1^)	948.67 ± 283.90	1108.33 ± 61.48	941.33 ± 168.15	1122.33 ± 31.83	1063.50 ± 76.42

* VS normal saline (P < 0.05), and

# *VS solvent (P < 0.05)*.

#### Clinical Chemistry

In male rats, clinical chemistry examination showed the following differential results (*P* < 0.05). ALT was higher in the high dose group than in the saline group. LDH was significantly higher in the high, medium, low, and solvent groups than in the saline group. UA was significantly higher in both the high and low dose groups than in the saline group. GLU was significantly higher in the solvent group than in the rest of the test groups, and there were no significant differences between the other test groups. Ivermectin microemulsion also caused alterations in some clinical chemistry parameters in female rats. AST was significantly lower in the medium and low dose groups than in the solvent group and the low dose was significantly lower than in the saline group; Significantly lower TG in the high and medium dose groups than in the solvent group, but not significantly different from the saline group Both HDLC and LDLC were significantly lower in the high and medium dose groups than in the saline group (*P* < 0.05); TCH was also significantly lower in the high and medium dose groups than in the saline group (*P* < 0.05). The results of effects of ivermectin microemulsion on clinical chemistry in rats were shown in Tables 3 and 4.

**Table 3:** Effects of ivermectin microemulsion on clinical chemistry in male rats.

**Parameters**	**High dose**	**Medium dose**	**Low dose**	**Solvent**	**Normal saline**
TBIL (μ mol· L^−1^)	0.43 ± 0.15	0.56 ± 0.15	0.58 ± 0.15	0.57 ± 0.23	0.64 ± 0.26
DBIL (μ mol· L^−1^)	2.60 ± 1.03	2.14 ± 0.73	2.30 ± 0.69	2.35 ± 0.88	1.76 ± 1.08
TP (g·L^−1^)	57.45 ± 9.77	55.74 ± 4.13	53.95 ± 8.11	54.76 ± 6.43	48.15 ± 1.98
ALB (g·L^−1^)	26.38 ± 4.83	28.24 ± 5.11	26.40 ± 5.04	27.03 ± 7.06	25.74 ± 6.61
ALT (U·L^−1^)	71.53 ± 15.39^*^	53.82 ± 11.24	49.83 ± 16.16	62.72 ± 15.19	45.96 ± 17.13
AST (U·L^−1^)	192.50 ± 17.99	196.50 ± 63.32	168.20 ± 77.33	200.24 ± 24.90	143.60 ± 19.74
ALP (U·L^−1^)	127.50 ± 168.84	135.40 ± 21.93	137.17 ± 15.99	133.67 ± 19.63	140.60 ± 26.29
LDH (U·L^−1^)	2604.50 ± 676.85^*^	2584.20 ± 447.19^*^	2403.50 ± 465.40^*^	2701.16 ± 488.95^*^	1684.80 ± 339.30
CK (U·L^−1^)	1739.35 ± 258.46	1731.32 ± 535.99	2263.66 ± 774.70	2010.78 ± 654.81	2436.88 ± 258.66
BUN (m mol· L^−1^)	7.95 ± 0.87	7.22 ± 1.21	8.63 ± 0.79^*^	7.63 ± 1.34	6.42 ± 1.81
CREA (μ mol· L^−1^)	59.63 ± 14.66	46.50 ± 6.38	44.37 ± 6.82	46.77 ± 8.67	49.54 ± 10.23
UA (μ mol· L^−1^)	91.00 ± 16.34^*^	71.96 ± 15.78	71.17 ± 9.92^*^	69.30 ± 20.09	38.80 ± 7.75
GLU (m mol· L^−1^)	7.30 ± 1.54^#^	8.12 ± 1.18^#^	7.55 ± 2.25^#^	11.08 ± 2.23^*^	7.96 ± 1.03
TG (m mol· L^−1^)	1.16 ± 0.24	1.71 ± 0.40	1.55 ± 0.34	1.03 ± 0.67^*^	1.79 ± 0.94
HDLC (m mol· L^−1^)	0.57 ± 0.11	0.61 ± 0.15	0.62 ± 0.14	0.59 ± 0.15	0.48 ± 0.13
LDLC (m mol· L^−1^)	0.23 ± 0.07	0.21 ± 0.08	0.26 ± 0.07	0.24 ± 0.13	0.22 ± 0.13
TCH (m mol· L^−1^)	1.41 ± 0.25	1.73 ± 0.45	1.90 ± 0.24	1.57 ± 0.58	1.63 ± 0.68
HBDH (U·L^−1^)	961.55 ± 483.50	959.70 ± 355.69	930.80 ± 218.80	962.73 ± 478.96	533.88 ± 45.71

* VS normal saline (P < 0.05), and

# *VS solvent (P < 0.05)*.

**Table 4:** Effects of ivermectin microemulsion on clinical chemistry in female rats.

**Parameters**	**High dose**	**Medium dose**	**Low dose**	**Solvent**	**Normal saline**
TBIL (μ mol· L^−1^)	0.50 ± 0.08	0.57 ± 0.12	0.58 ± 0.16	0.58 ± 0.15	0.56 ± 0.09
DBIL (μ mol· L^−1^)	1.85 ± 0.59	1.35 ± 0.37	1.83 ± 0.69	2.10 ± 0.30	1.74 ± 1.34
TP (g·L^−1^)	48.18 ± 3.73	52.82 ± 4.22	54.27 ± 3.78	51.93 ± 10.29	56.42 ± 12.95
ALB (g·L^−1^)	24.93 ± 3.53	23.85 ± 2.73	23.72 ± 5.39	23.90 ± 2.89	24.08 ± 8.62
ALT (U·L^−1^)	48.68 ± 19.88	40.05 ± 8.09	44.02 ± 14.22	57.20 ± 11.60	47.66 ± 26.97
AST (U·L^−1^)	106.00 ± 9.16	105.20 ± 14.50^#^	84.67 ± 12.06^#*^	140.50 ± 45.44	143.50 ± 10.47
ALP (U·L^−1^)	148.00 ± 21.00	144.20 ± 31.22	125.17 ± 24.63	149.33 ± 48.02	115.60 ± 42.48
LDH (U·L^−1^)	1960.75 ± 544.18	1777.67 ± 468.03	2279.67 ± 606.47	2337.83 ± 698.37	1875.80 ± 391.69
CK (U·L^−1^)	2714.53 ± 298.91	2173.15 ± 653.68	2663.07 ± 535.62	2228.40 ± 674.23	2771.58 ± 173.69
BUN (m mol· L^−1^)	8.48 ± 1.71	7.20 ± 0.76	7.55 ± 1.55	8.20 ± 1.58	7.24 ± 1.97
CREA (μ mol· L^−1^)	42.13 ± 18.24	35.97 ± 6.84	39.07 ± 10.16	39.05 ± 7.74	41.62 ± 7.02
UA (μ mol· L^−1^)	61.63 ± 15.08	47.23 ± 11.25	70.17 ± 18.45	71.77 ± 22.07	46.08 ± 10.98
GLU (m mol· L^−1^)	7.013 ± 1.148	7.667 ± 0.922	7.227 ± 1.860	8.044 ± 1.908	7.791 ± 1.616
TG (m mol· L^−1^)	1.03 ± 0.32^#^	1.30 ± 0.20^#^	1.60 ± 0.27	1.96 ± 0.79	1.45 ± 0.50
HDLC (m mol· L^−1^)	0.35 ± 0.11^#*^	0.50 ± 0.12^*^	0.57 ± 0.12	0.62 ± 0.12	0.71 ± 0.18
LDLC (m mol· L^−1^)	0.22 ± 0.06^*^	0.22 ± 0.08^*^	0.31 ± 0.13	0.32 ± 0.07	0.39 ± 0.16
TCH (m mol· L^−1^)	1.32 ± 0.23^*^	1.46 ± 0.28^*^	1.85 ± 0.42	2.12 ± 0.37	1.82 ± 0.50
HBDH (U·L^−1^)	759.48 ± 221.51	650.22 ± 184.72	877.37 ± 282.21	917.67 ± 324.16	857.92 ± 436.60

* VS normal saline (P < 0.05), and

# *VS solvent (P < 0.05)*.

### Organ Coefficient and Histopathology

#### Organ Coefficient

The liver coefficients of male rats in the high dose, medium dose, and solvent groups were significantly elevated compared to the saline group (*P* < 0.05), whereas the liver coefficients of male rats in the low dose group were not significantly different from the saline group (*P* > 0.05). The brain coefficients of male rats in the medium dose group were significantly greater than those in the saline group. The effect of ivermectin microemulsion on organ coefficients appears to be more pronounced in female rats, as follows, the heart coefficient was greater in the medium dose group than in the saline group (*P* < 0.05); The liver coefficient was significantly higher in the high and medium dose groups than in the saline group (*P* < 0.05); The kidney coefficient was statistically higher in the high dose group than in the saline group (*P* < 0.05); The ovarian coefficients in the high and medium dose groups were not different from those in the solvent group (*P* > 0.05), but were significantly lower than those in the saline group (*P* < 0.05); Brain coefficients were significantly increased in the high, medium, and low dose groups compared to the solvent group (*P* < 0.05), but were not significantly different from the saline group (*P* > 0.05). The effects of ivermectin microemulsion on coefficient of organs in rats as shown in Tables 5 and 6.

**Table 5:** Effects of ivermectin microemulsion on coefficient of organs in male rats.

**Organs**	**High dose**	**Middle dose**	**Low dose**	**Solvent**	**Normal saline**
Heart	0.0038 ± 0.0003	0.0038 ± 0.0002	0.0039 ± 0.0004	0.0044 ± 0.0010	0.0040 ± 0.0003
Liver	0.0360 ± 0.0005^*^	0.0367 ± 0.0037^*^	0.0318 ± 0.0021^#^	0.0383 ± 0.0048^*^	0.0285 ± 0.0030
Spleen	0.0022 ± 0.0002	0.0022 ± 0.0001	0.0022 ± 0.0001	0.0021 ± 0.0002	0.0021 ± 0.0002
Lung	0.0072 ± 0.0009	0.0066 ± 0.0014	0.0070 ± 0.0011	0.0069 ± 0.0010	0.0073 ± 0.0012
Kidney	0.0069 ± 0.0005	0.0070 ± 0.0006	0.0068 ± 0.0005	0.0069 ± 0.0005	0.0069 ± 0.0004
Testis	0.0042 ± 0.0011	0.0091 ± 0.0044	0.0124 ± 0.0005	0.0117 ± 0.0005	0.0121 ± 0.0010
Brain	0.0065 ± 0.0008	0.0067 ± 0.0008^*^	0.0038 ± 0.0004	0.0052 ± 0.0016	0.0051 ± 0.0017

* VS normal saline (P < 0.05), and

# *VS solvent (P < 0.05)*.

**Table 6:** Effects of ivermectin microemulsion on coefficient of organs in female rats.

**Organs**	**High dose**	**Middle dose**	**Low dose**	**Solvent**	**Normal saline**
Heart	0.0040 ± 0.0004	0.0044 ± 0.0007^*^	0.0038 ± 0.0004	0.0039 ± 0.0003	0.0037 ± 0.0003
Liver	0.0331 ± 0.0020^*^	0.0325 ± 0.0011^*^	0.0311 ± 0.0033	0.0287 ± 0.0017	0.0292 ± 0.0015
Spleen	0.0021 ± 0.0001	0.0021 ± 0.0002	0.0022 ± 0.0003	0.0020 ± 0.0002	0.0022 ± 0.0003
Lung	0.0056 ± 0.0003	0.0059 ± 0.0007	0.0073 ± 0.0012	0.0064 ± 0.0001	0.0060 ± 0.0003
Kidney	0.0069 ± 0.0004^*^	0.0065 ± 0.0003	0.0062 ± 0.0009	0.0064 ± 0.0010	0.0062 ± 0.0003
Ovary	0.0042 ± 0.0011^#*^	0.0041 ± 0.0004^#*^	0.0069 ± 0.0009	0.0067 ± 0.0004	0.0070 ± 0.0007
Brain	0.0081 ± 0.0007^#^	0.0081 ± 0.0002^#^	0.0078 ± 0.0004^#^	0.0064 ± 0.0002^*^	0.0083 ± 0.0002

* VS normal saline (P < 0.05), and

# *VS solvent (P < 0.05)*.

#### Histopathology

**Figure 2:**
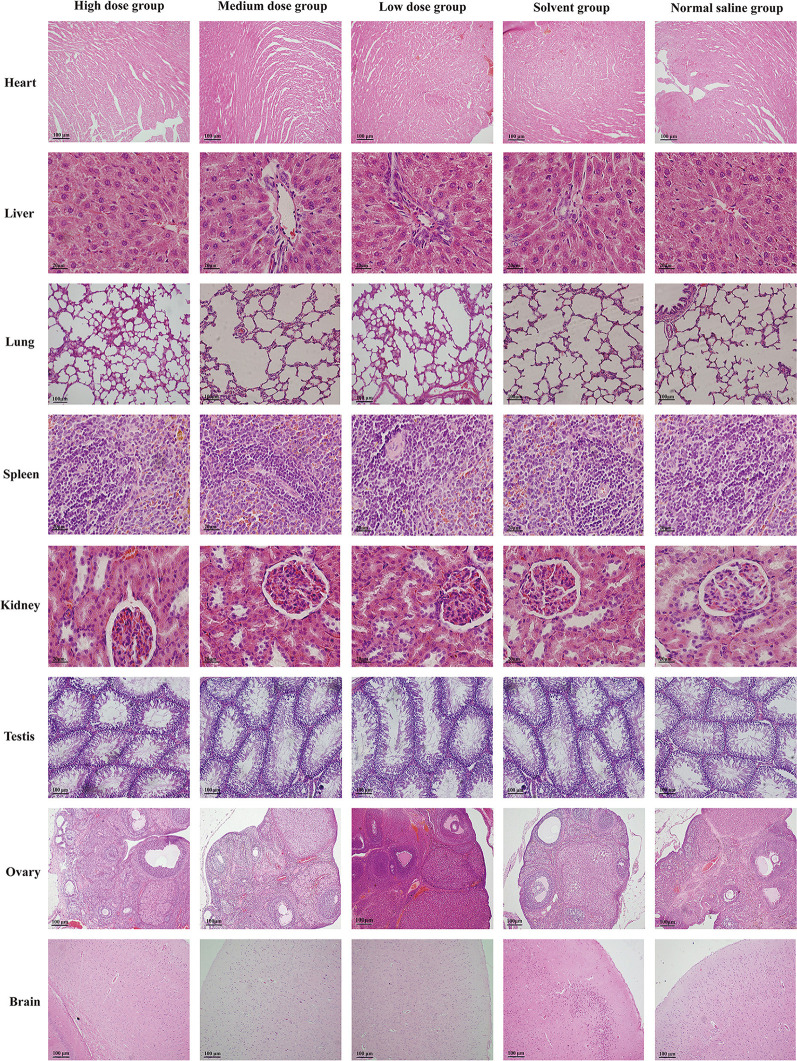
Photomicrographs histopathological observation on organs of rats in each group. H&E stain, original magnification × 100.

Under light microscope, there was no obvious abnormality in the tissue structure of heart, liver, spleen, lung, kidney, ovary, and testis compared with normal saline and solvent group. The myocardial fibers were columnar branches and arranged neatly. The hepatocytes are arranged neatly to form a hepatic cord around the central vein, and the interlobular artery and interlobular bile duct can be seen in the hepatic portal area. The trabeculae formed by connective tissue can be seen extending into the white pulp in the spleen. The structure of the lung is clear, and terminal bronchioles, respiratory bronchioles, alveolar ducts, and alveolar sacs can be seen. The vascular ball formed by capillaries can be seen in the renal cortex, and the proximal convoluted tubule is cuboidal epithelium with brush margin; the cuboidal epithelial cells of distal convoluted tubule are neatly arranged, but there is no obvious brush margin. The brain glia was fine and full, and the neuron-shaped structure was not abnormal. The section of the ovary is oval, the external reproductive epithelium is irregular, there are follicles at different developmental stages, the deep surface of the white membrane is the cortex and medulla, the cortex is in the periphery, and the medulla is in the center of the ovary, which is composed of loose connective tissue and contains many large blood vessels. The lumen of seminiferous tubule was regular in testicular tissue. Sertoli cells, spermatogonia, primary spermatocytes, and spermatozoa were found in the lumen, and there were interstitial cells in the interstitial of seminiferous tubule. The results of histopathological examination of the organs of rats in each group are shown in Figure 2.

## Discussion

Ivermectin is a widely used broad-spectrum antiparasitic drug, which has a good killing effect on both endoparasites and ectoparasites. In order to improve solubility and provide bioavailability, we have developed a novel preparation, ivermectin microemulsion. The purpose of this study was to clarify the toxic effect of ivermectin microemulsion, because there was no previous longer-term toxicity test of ivermectin microemulsion.

After intraperitoneal injection for 14 days, it was found that there were slight clinical reactions such as dull, curled up and reduced feed intake in the high dose group at the beginning of administration, and the symptoms disappeared after 4 days. On the 7th day, the mean weight of the high dose group was slightly lower than that of the normal saline group, but there was no significant difference between the high dose group and the normal saline group. The intake and drinking water of rats in other dose groups were normal. On the 14th day of administration, there was no significant difference in weight gain among each dose group. At the initial stage of administration, it has stress or slight toxic effect on the rats in the high dose group, but it can recover after a few days, indicating that the toxic effect is non-permanent and reversible. There were no significant differences between males and females, and they showed the same trend.

Hematological testing of rodents is recognized as an integral part of toxicity and safety assessment (13, 14). Slight pathological changes of the body, tissues or organs can cause changes in blood composition, so the results of hematology examination are of great help to understand the diseases of the body (15, 16). In this experiment, both male and female rats showed leucocytosis in high dose group (*P* < 0.05), in addition to this, reductions in RBC, HGB and HCT were also found in female rats in the high dose group. The results point to the fact that high doses of ivermectin microemulsion can cause hematological fluctuations. Some studies have suggested that the observed alterations in hematopoietic system parameters may indicate interference with the hematopoietic function of the bone marrow system by exogenous substances (17, 18). Studies on ivermectin have shown that it does cause a decrease in hemoglobin levels, but the results are not significant (19). In addition, overnight feeding may also lead to a decrease in leukocytes, blood glucose, BUN, ALT, and ALP in rats (20). Although there is insufficient evidence to prove that the decline in hematological parameters is due to ivermectin suppression of the hematopoietic system, it is still noteworthy. From the results of hematological examination, female rats were more sensitive to the toxicity of ivermectin microemulsion.

Male and female rats showed different changes after the injection of ivermectin microemulsion. In male rats, high doses of ivermectin microemulsion caused elevations in ALT, LDH, and UA. The liver and kidney are the most sensitive predictors of chemical toxicity in the mammalian system and are well-characterized by histopathology and serum biochemistry (21). Serum ALT and AST are the most used biomarkers in the detection of liver function and integrity and are considered sensitive indicators of hepatic chemically induced injury (22, 23). Previous studies have reported elevated ALT and AST at therapeutic doses of ivermectin in rats (24). In addition, controlling mice by grasping the abdomen or injecting drugs may result in an elevation of serum ALT, which may be due to mechanical damage to the liver (25). Liver and skeletal muscle are considered to be the main sources of LDH (26). All four treatment groups except the saline group had similarly increased serum LDH activity, and there was no significant difference between the 4 groups. This seems to point out the irritating effect of the solvent on the muscles. Previous laboratory results have also demonstrated that ivermectin microemulsion injections are extremely mildly irritating (12). Unpredictably, male rats had elevated UA in both high and low doses and showed non-dose correlation. Serum uric acid is also considered a marker of tubular reabsorption and “efficient” circulating blood volume (27). The elevated UA may be due to increased activity of LDH and consequently increased pyruvate. The ketoacids, which are important inhibitors of the anion transport system, lead to the inhibition of tubular secretion (28). It was not possible to demonstrate a toxic effect of ivermectin microemulsion on kidney, as the more sensitive parameters, BUN and CREA, did not show significant changes. The reduction in AST present in female rats was considered to be of no toxicological significance. Reduced TG (both HDLC and LDLC) and TCH were seen in female rats. Mild or moderate increases or decreases in serum cholesterol and/or triglyceride concentrations are relatively common in toxicology studies, and the exact mechanisms involved are often unknown (20).

After excluding the influence of other interference factors such as water loss before weighing, the organ coefficient increased, indicating that the organs of the experimental animals had changes such as hyperaemia, oedema and hypertrophy, and the organ coefficient decreased. It shows that the organs of experimental animals may have atrophy, degeneration and other changes (29, 30). Schauss et al. (31) pointed out that whether the change of organ weight has toxicological significance should be combined with the results of histomorphology and pathological examination. Male rats in the high and medium dose and solvent groups had significantly higher hepatic coefficients than those in the low dose and saline groups. This change also occurred in female rats. The remaining alterations in the organ coefficients could not be linked to a possible biological significance. However, it is notable that the ovarian coefficient was significantly reduced in the high and medium dose groups of rats. High doses of ivermectin have been reported to be teratogenic to the fetus, and it can also induce apoptosis in uterine lumen epithelial (pLE) cells (32). But there is no information yet on the effects of ivermectin on the ovaries.

The hematology and biochemical indexes are important basis for judging the health status, while histopathology go deep into the level of tissue or cell to further intuitively judge the health status of animals. The pathological examination of tissues and organs is helpful to the diagnosis of diseases, which is not only the golden index for the diagnosis of most diseases, but also the direct evidence that drug toxicity may cause damage to the body (20). Histopathological examination was carried out on 8 main organs of rats. The results showed that the tissue structures of heart, liver, spleen, lung, kidney, brain, ovary, and testis in each dose group were clear, and there were no obvious pathological changes.

According to the previous acute toxicity results of the laboratory, LD_50_ of ivermectin microemulsion on rats by intraperitoneal injection is 3.825 g/kg. The LD_50_ of direct injection of ivermectin in rats is 51.5 mg/kg (33). Compared with the API (Active Pharmaceutical Ingredient), the acute toxicity of ivermectin microemulsion was reduced by about 70×. Lei et al. (34) reported that the subacute inhalation toxicity test of ivermectin in rats showed that the liver may be the target organ of toxicity of ivermectin. The results of oral subchronic test showed that higher doses ivermectin had some effect on the liver, kidney, spleen, and bodyweight (35). In addition, other studies have shown that testis and epidermis have reproductive toxicity when the dose is more than 3 mg/kg; testicular coefficient and epidermis effect (36). In this experiment, high and medium doses (especially high doses) had a potential risk of disturbing the hematopoietic system, with females having a higher sensitivity. Elevated activity of serum ALT and LDH also pointed to a potential risk of liver injury, whereas increased UA was an indirect effect. The changes in cholesterol and triglycerides in female rats due to high doses of the drug may reflect metabolic or adaptive changes that are less relevant to potential toxicity than the reduced erythroid lineage parameters and increased ALT associated with the tested drug (20). The changes that occurred at the hematological and clinical chemistry levels did not correspond in the histopathological examination, indicating that ivermectin microemulsion injections had little effect on tissue levels. In addition, although ovarian tissue did not reveal drug-related toxic pathology at the microscopic level, the reduction in the organ coefficient is still noteworthy. At low doses, ivermectin microemulsion injections did not exhibit significant, systemic, and dose-related toxicities.

## Conclusion

The rats in low dose group did not show test-related toxicity indications. Ivermectin microemulsion injections at low doses for longer periods of time have a good safety profile. Therefore, it can be considered that the NOVEL of ivermectin microemulsion injection is 0.19 g/kg. The experimental results can provide support and reference for the safe use of ivermectin microemulsion in clinic.

## Data Availability Statement

The original contributions presented in the study are included in the article/supplementary material, further inquiries can be directed to the corresponding authors.

## Ethics Statement

The animal study was reviewed and approved by Animal Ethics Committee of Lanzhou Institute of Husbandry and Pharmaceutical Science.

## Author Contributions

Conceptualization: ZD and J-yZ. Methodology: ZD and S-yX. Software: S-yX. Validation: X-zZ. Sample processing: S-yX and ZD. Resources: J-yZ. Data curation: ZD. Writing—original draft preparation: S-yX and ZD. Writing—review and editing: ZD. Visualization: S-yX. Project administration: X-zZ. Funding acquisition: J-yZ. All authors read and approved the final manuscript.

## Conflict of Interest

S-yX was employed by the company China Agricultural Vet. Bio. Biomedical Co., Ltd. The remaining authors declare that the research was conducted in the absence of any commercial or financial relationships that could be construed as a potential conflict of interest.
